# Case report: Braid-like right coronary artery with chest pain

**DOI:** 10.3389/fcvm.2023.1225020

**Published:** 2023-06-28

**Authors:** Wenbo Wang, Lan Lan, Huijuan Hu

**Affiliations:** Department of Radiology, Zhongnan Hospital of Wuhan University, Wuhan, China

**Keywords:** braid-like coronary artery, woven coronary artery, coronary variant, coronary angiography, thrombus recanalization, coronary artery dissection introduction

## Abstract

Braid-like coronary artery is very rare. It is featured by the division of the coronary artery into multiple tortuous small channels that later converge into a normal lumen at any segment of the coronary artery. We presented a case of a 27-year-old male patient with occasional chest pain. In coronary CT angiography (CCTA) and coronary angiography, a braid-like appearance was found in the right coronary artery. After multidisciplinary discussion, it was speculated to be a woven coronary artery (WCA). We conducted a literature review about woven coronary arteries.

## Introduction

Braid-like coronary artery is extremely rare. In these cases, the coronary artery is divided into multiple thin channels at any segment of the coronary artery, and subsequently, these channels merge again into a normal conduit (1). Up to now, only a limited number of cases have been reported. Woven coronary artery (WCA) and other diseases such as revascularization thrombosis or coronary artery dissection can cause braid-like changes (1–3). In the current case report, we describe a 27-year-old male patient with a braid-like right coronary artery.

## Case presentation

A 27-year-old man visited our cardiology department with needle-like chest pain after engaging in energetic exercise on 20 Feb 2023. One year previously, right coronary artery calcification had been found in a routine chest CT examination. During the one-year period since the discovery of right coronary artery calcification, the patient had occasional chest pain after vigorous exercise, usually lasting for a few minutes and that relieved itself, without radiating pain in other parts. In order to perform further diagnosis and treatment, the patient was hospitalized. On admission, his blood pressure was 127/88 mmHg, heart rate was 90 bpm. The body mass index was 24.3. He had no history of any cardiovascular disease risk factors. He denied any relevant family history or smoking history. He also denied the use of medication and excessive alcohol use.

Cardiac-related biochemical indices were normal as follows: N-terminal pro-brain natriuretic peptide (NT-Pro BNP) was 13.2 pg/ml (normal range 0–450 pg/ml), creatinine kinase (CK) was 89 U/L (normal range <171 U/L), CK-MB was 14 U/L (normal range 0–25 U/L), and high sensitivity cardiac troponin was 1.9 pg/ml (normal range 0–26.2 pg/ml). Other biochemical indications and blood routine examinations were also normal.

**Figure 1:**
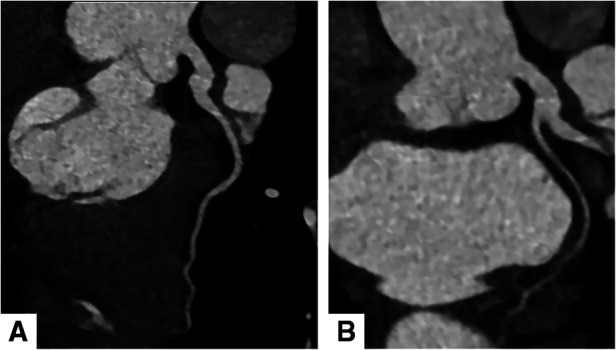
Computed tomography coronary angiography showed normal left anterior descending artery (**A**) and left circumflex artery (**B**).

**Figure 2:**
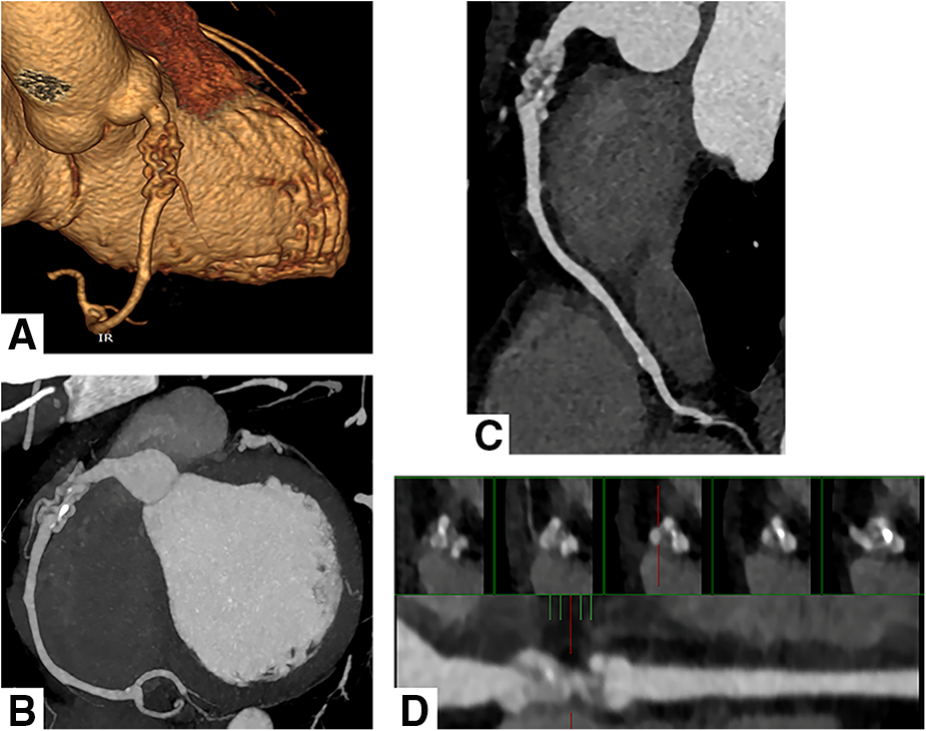
Computed tomography coronary angiography shows the WCA in the right coronary artery. Volume rendering reconstruction (**A**), Maximum intensity projection (**B**) and Curved multiplanar reconstruction (**C**) revealed multiple twisting channels in the proximal segment of the right coronary artery and merged again into a normal conduit. Small patchy calcification was observed in the structure. (**D**) Consecutive cross-sectional images at the lesion site showed twisting and multiple small channels.

**Figure 3:**
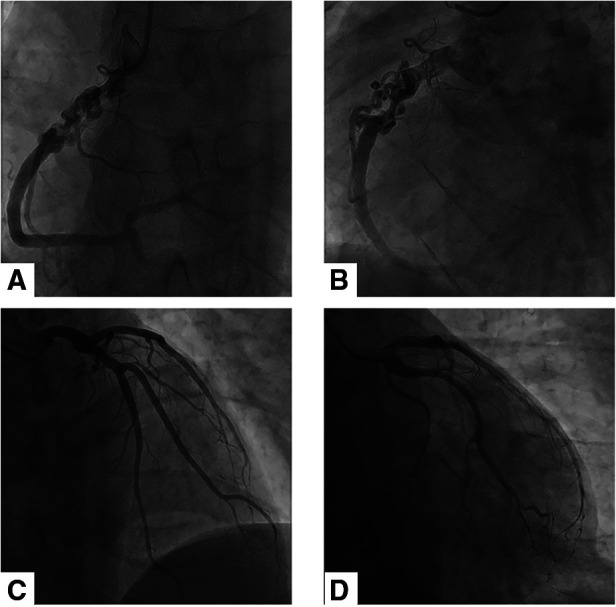
Coronary angiographic findings of the patient. (**A**) and (**B**) showed the braid-like change at the proximal portion of RCA. There was a normal blood flow in the distal segment of the anomaly. (**C**) and (**D**) revealed the normal left anterior descending artery and left circumflex artery.

Electrocardiography showed sinus rhythm. Transthoracic echocardiography (TTE) indicated normal cardiac structure and ventricular function, with a normal ejection fraction of 65%. Computed tomography coronary angiography (CTCA) showed that there was no lumen stenosis in the left anterior descending artery or left circumflex artery (Figures 1A,B). However, a braid-like change was shown at the proximal portion of the right coronary artery (RCA). The vessel was divided into multiple small channels with a slightly distorted course along a 2 cm length of segment, subsequently, the channels merged again into a normal lumen. Patchy calcification was found in the structure (Figures 2A–D). In order to further clarify the possible etiology, the patient underwent coronary angiography (CAG), which demonstrated the same imaging findings as the CTCA (Figures 3A–D). Distal to the woven segment, the downstream blood flow was normal with a thrombolysis in myocardial infarction III (TIMI-III) grade blood flow. Unfortunately, because of the small diameter, the patient did not undergo intravascular ultrasound (IVUS) and optical coherence tomography (OCT) examination.

The case was discussed in the cardiac multi-disciplinary team. Woven coronary artery, intracoronary thrombus, and spontaneous coronary artery dissection can mimic the braid-like appearance seen in the patient. Combined with the patient's medical history and imaging characteristics, the RCA lesion was speculated as a woven coronary artery. The patient rejected the suggestion to undergo percutaneous coronary intervention (PCI). He was given antiplatelet and antithrombotic agents (aspirin 100 mg qd and clopidogrel bisulfate tablet 75 mg qd), with a cholesterol-lowering drug (atorvastatin 10 mg qn). The patient was advised to have check-ups regularly to review the situation and that, if the symptoms worsened, surgery should be considered. On May 18, a telephone follow-up was performed, and the patient was in good condition.

## Discussion

Braid-like coronary artery is an uncommon illness, which is marked by the division of the coronary artery into multiple tortuous small channels, which, after some distance, merge again into a normal lumen. The numerous small tortuous channels resemble braided hair. WCA was first described by Sane and Vidaillet in 1988, and they defined it as an extremely rare and benign congenital anomaly (1). Apart from WCA, coronary artery dissection (CAD) or revascularization thrombosis can also cause braid-like change (1–3). It is very important to make a differential diagnosis and provide some information about the underlying etiology for clinicians to make optimal treatment decisions. When making the differential diagnosis, there are some important points. First, generally speaking, artery dissection is more likely to occur in a larger vessel such as the aorta than in a smaller vessel such as a coronary artery. Second, almost all patients with CAD present with acute coronary syndrome and elevation of cardiac enzymes (4). Third, CAD usually demonstrates poor flow due to the effect of the false lumen (5). In this case, the patient was in stable condition, and the cardiac-related biochemical indices were normal, combined with the normal downstream blood flow distal to the woven segment, therefore CAD was excluded. But the differential diagnosis between revascularization thrombosis and WCA was very challenging. In the case of revascularization thrombosis, the blood flow should be reduced because of the intraluminal extension of the thrombus (6). WCA was previously thought to be a benign anomaly. Now, however, the thrombus or atherosclerosis caused by woven coronary arteries has been reported because of the distorted thin channels (5, 7). IVUS and OCT were helpful for differential diagnosis (8). In woven coronary arteries, OCT findings demonstrate multiple small channels separated by fibrous tissue with no cross-communication between those channels; each channel contains a relatively complete three-layer vascular structure (8, 9). It is a pity that the patient did not undergo IVUS or OCT examination. Yet the downstream blood flow distal to the woven segment was normal, so the patient was inferred to be a WCA case.

WCA is an exceptionally rare congenital variation. Because of the rarity, the data on this anomaly are limited. Bamousa B et al. (10) reported that there were only 37 WCA cases from 1998 to June 2021. Most patients were adults except for a 9-month-old infant with Kawasaki disease (7). With regard to this anomaly, male patients were predominant, with a male-to-female ratio of 10:1. RCA was the most common vessel of involvement (70.2%) and LAD came second (32.4%), followed by LCX (18.9%). Nevertheless, cases have been reported in two or three coronary arteries simultaneously (10). It can occur in any segment of the vessel, whereas the most common site is the proximal segment (5, 11, 12). The average length affected by WCAA was 2.2 cm (5, 12–14).

The etiology of woven coronary artery is currently not well understood. Therapies vary from conservative medical treatment to bypass surgery. Generally, WCA is regarded as benign and asymptomatic, and no treatment is required. However, as the number of reports increased, there have been some cases of WCA with malignant cardiac events such as thrombus formation, atherosclerosis, angina, acute coronary syndrome, myocardial infarction, chronic ischemia, and even sudden cardiac death (3, 14–19). The main reason for this is that the woven-like structure interferes with the laminar flow in the coronary arteries and increases the shear forces on the arterial wall. Finally, it expedites the process of coronary atherosclerosis and life-threatening situations (20). By using hydromechanics principles, Cito S et al. (21) put forward that the morphology and underlying physiopathology of WCA could cause coronary pressure loss. The greater the length and the number of channels, the more the pressure will drop. Therefore, if coronary atherosclerosis or thrombosis is observed and the patient has symptoms, the presence or absence of myocardial ischemia needs to be confirmed before treatment. Coronary artery bypass grafting may be the best therapy choice because stent implantation may be difficult in the small channels of the WCA and have a greater risk of complications (8). In our case, we hypothesized that the patient's chest pain after vigorous exercises was attributable to coronary calcification and coronary pressure loss. PCI was recommended, but the patient refused.

In conclusion, WCA is a very uncommon congenital coronary variation. It is not just a benign lesion as some adverse cardiovascular events have been reported. In the future, when woven-like coronary change is encountered, the collection of detailed medical history, multimodality imaging examination, especially IVUS and OCT, and long-time follow-up should be emphasized.

## Data Availability

The original contributions presented in the study are included in the article, further inquiries can be directed to the corresponding author.
